# Random forests algorithm using basic medical data for predicting the presence of colonic polyps

**DOI:** 10.3389/fsurg.2025.1523684

**Published:** 2025-03-03

**Authors:** Mihaela-Flavia Avram, Nicolae Lupa, Dimitrios Koukoulas, Daniela-Cornelia Lazăr, Mihaela-Ioana Mariș, Marius-Sorin Murariu, Sorin Olariu

**Affiliations:** ^1^ Department of Surgery X, 1st Surgery Discipline, “Victor Babeș” University of Medicine and Pharmacy Timișoara, Timisoara, Romania; ^2^ Abdominal Surgery and Phlebology Research Center, “Victor Babes” University of Medicine and Pharmacy, Timisoara, Romania; ^3^ Department of Mathematics, “Politehnica” University of Timişoara, Timisoara, Romania; ^4^ Department of Gastroenterology, Municipal Hospital “Dr. Teodor Andrei”, Lugoj, Romania; ^5^ Department V of Internal Medicine I, Discipline of Internal Medicine IV, “Victor Babeș” University of Medicine and Pharmacy, Timisoara, Romania; ^6^ Department of Functional Sciences, Pathophysiology, “Victor Babes” University of Medicine and Pharmacy, Timisoara, Romania; ^7^ Center for Translational Research and Systems Medicine, “Victor Babes” University of Medicine and Pharmacy, Timisoara, Romania

**Keywords:** colorectal polyps, random forests, machine learning, colorectal cancer prevention, risk prediction model, artificial intelligence

## Abstract

**Background:**

Colorectal cancer is considered to be triggered by the malignant transformation of colorectal polyps. Early diagnosis and excision of colorectal polyps has been found to lower the mortality and morbidity associated with colorectal cancer.

**Objective:**

The aim of this study is to offer a predictive model for the presence of colorectal polyps based on Random Forests machine learning algorithm, using basic patient information and common laboratory test results.

**Materials and methods:**

164 patients were included in the study. The following data was collected: sex, residence, age, diabetes mellitus, body mass index, fasting blood glucose levels, hemoglobin, platelets, total, LDL and HLD cholesterol, triglycerides, serum glutamic-oxaloacetic transaminase, chronic gastritis, presence of colonic polyps at colonoscopy. 80% of patients were included in the training set for creating a Random forests algorithm, 20% were in the test set. External validation was performed on data from 42 patients. The performance of the Random Forests was compared with the performance of a generalized linear model (GLM) and support vector machine (SVM) built and tested on the same datasets.

**Results:**

The Random Forest prediction model gave an AUC of 0.820 on the test set. The top five variables in order of importance were: body mass index, platelets, hemoglobin, triglycerides, glutamic-oxaloacetic transaminase. For external validation, the AUC was 0.79. GLM performance in internal validation was an AUC of 0.788, while for external validation AUC-0.65. For SVN, the AUC - 0.785 for internal validation and 0.685 for the external validation dataset.

**Conclusions:**

A random forest prediction model was developed using patient's demographic data, medical history and common blood tests results. This algorithm can foresee, with good predictive power, the presence of colonic polyps.

## 1 Introduction

Colorectal cancer (CRC) is the second cause of cancer related deaths worldwide, having a 4%–5% lifetime long risk of appearing in the general population. It is estimated that, in the absence of screening strategies, 7.7%–8.5% of persons above 40 years old would develop CRC and 3.2%–3.4% would die of it (1).

CRC incidence and mortality have a decreasing trend in the majority of European Countries, USA and big part of Asia-Pacific. The incidence in USA has decreased by more than 35% since screening programs have been used in the 1990's. The detection of premalignant lesions is an important objective in CRC screening as the removal of polyps during colonoscopy is efficient in reducing the incidence of CRC (2, 3).

Studies have shown that when the progression from polyp to CRC takes places its duration is 10.6–25.8 years (4, 5). Detection and resection of these polyps reduces the incidence of CRC.

Research done on colonoscopies report an incidence of polyps of 20%–53% in adults aged >50years, with a 9.7% incidence of advanced adenomas (defined as adenomatous polyps sized >10 mm or with villous characteristics or having high grade dysplasia). Meta-analysis of these studies (for patients >50years old) determined a global prevalence rate of 24% for polyps and the prevalence of advanced adenomas – 4.5% (6–8).

The age of the screening initiation is crucial for the efficiency and rentability of screening programs. Simulation analysis in USA, which were the basis of screening recommendations for CRC made by US Preventive Service Task Force and American Cancer Society, state that 45 years old is a better age to start screening, as opposed to 50 years, providing a more efficient balance of life-years gained from screening and colonoscopy burden (5, 9). Simulation modelling analysis for CRC, taking into consideration the incidence in the younger population, have determined the American Cancer Society to recommend CRC screening to be started at 45 years for individuals with a moderate risk for CRC (10). Other countries have adjusted the starting age for CRC: Germany reduced the age from 55 to 50 years (for men only), in England, the UK National Screening Committee recommends to reduce the age from 60 to 50 years (11).

Colonoscopy is the gold standard for the diagnosis and treatment of colorectal polyps. This intervention requires the existence of adequate medical facility and dedicated personnel, so the possibility of performing colonoscopies is limited, no matter how rich the medical system is. The aim of this study is to offer a predictive model for the presence of colorectal polyps using basic patient information and laboratory test results. This model can be used for selecting patients which have a high risk of being diagnosed with colorectal polyps and to be offered a colonoscopy, even if they are not at the starting age for CRC screening, thus reducing the incidence of CRC in the general population.

## 2 Materials and methods

### 2.1 Study design

Data from patients who underwent colonoscopies between January 2022 and February 2023 in one hospital, Municipal Hospital “Dr. Teodor Andrei” Lugoj, Romania, was extracted. For external validation of the algorithm data from patients who underwent colonoscopies between June 2022-June 2023 in an outpatient gastroenterology facility, “Dr.K.D.Medic” Clinic, Caransebes, Romania. The study was approved by the Local Ethics Board.

**Figure 1:**
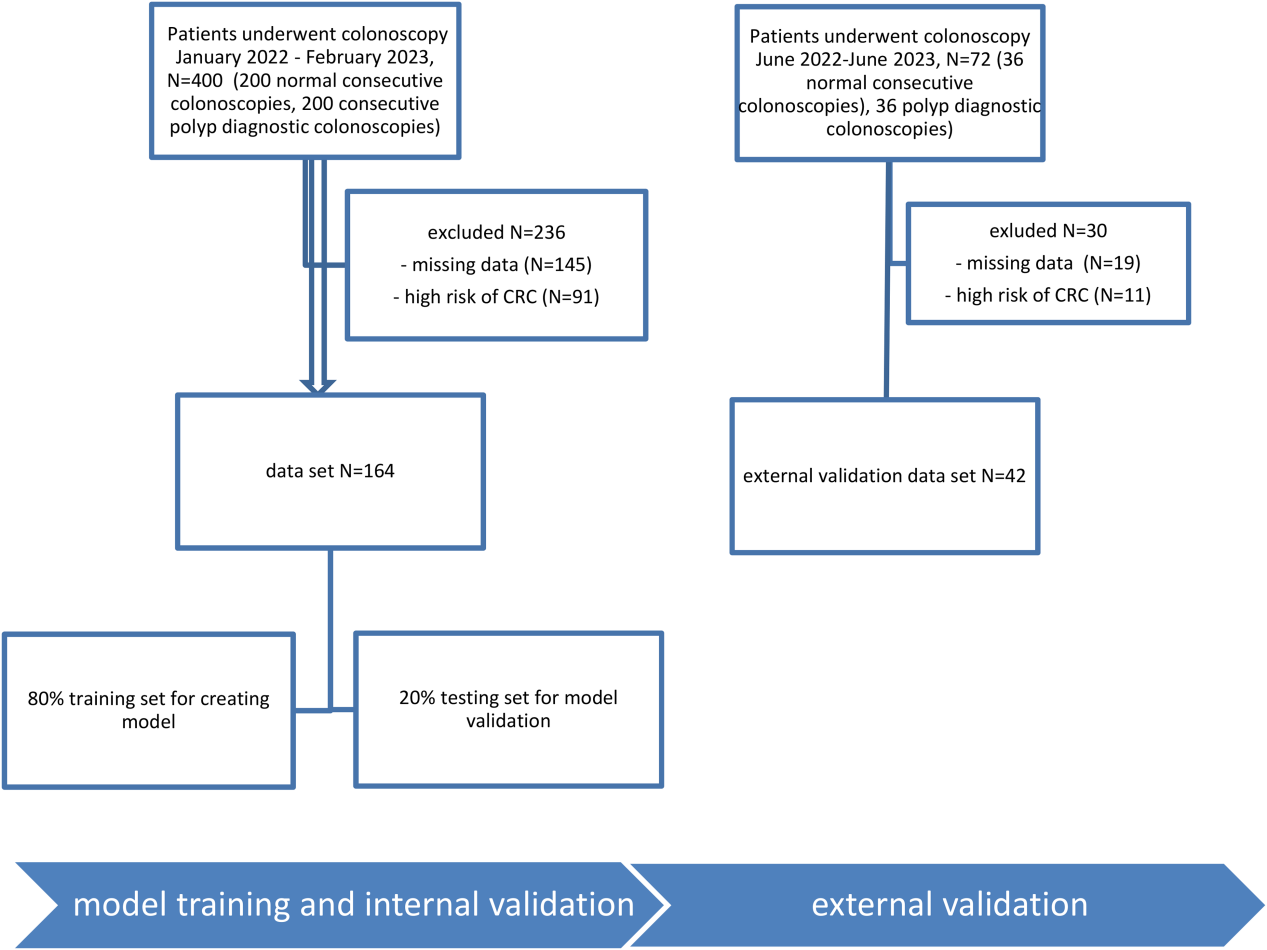
Patient selection.

Figure 1 shows patients' selection and analysis. 200 consecutive patients with normal colonoscopies and 200 consecutive patients with polyp diagnostic colonoscopies were selected from the medical records. Exclusion criteria were: missing data (145 patients) and patients with high risk of CRC (91 patients). The dataset included 164 patients which were randomly divided 80% into a training set for the development of the model and 20% into a test set for the validation of the model. The random forests were developed on the training set. The testing set was used to perform internal validation of the model created. The dataset for external validation selected 42 patients out of 72, as exclusion was done for 30 (19 – data was missing and 11 – high risk of CRC).

### 2.2 Data collection

The data included in this study was selected to include medical information that can be easily obtained in most adults (demographic data, medical history, common blood tests results not older than 12 months), the reason being to create a model which can be easily employed for future patients, which requires no additional costs.

Data collected from the patients' records, as seen in Table 1, included: sex (male/female), residence (urban or rural), age, diabetes mellitus (present or absent), BMI value (body mass index), fasting blood glucose levels, hemoglobin levels, platelets values, total cholesterol, LDL cholesterol, HDL cholesterol, triglycerides, serum glutamic-oxaloacetic transaminase, chronic gastritis (present or absent), the presence or absence of colonic polyps at colonoscopy.

**Table 1:** Data collected, variables and their abbreviation.

Variable	Values	Abbreviation in dataset
Sex	Male/female	Sex (male = 1, female = 2)
Residence	Urban/rural	Res (urban = 1, rural = 2)
Age	In years	Age
Diabetes mellitus	Absent/present	DZ (absent = 0, present = 1)
Body mass index	Normal weight: BMI 18.5–24.9.	IMC
Fasting blood glucose level	Normal values: 70–110 mg/dl	glic
Hemoglobin	Normal values:12–17 g/dl	Hb
Platelets	Normal values: 200–400 × 10^9^/L	plt
Total cholesterol	Normal values: 150–200 mg/dl	colest
LDL cholesterol	Normal values: 70–130 mg/dl	LDL
HDL cholesterol	Normal values: 44–80 mg/dl	HDL
Triglycerides	Normal values: 40–160 mg/dl	triglic
Serum glutamic-oxaloacetic transaminase	Normal values: 3–31 UI/L	TGO
Chronic gastritis	Absent/present	gastr (absent = 0, present = 1)
Colonic polyps	Absent/present	polip (absent = 0, present = 1)

### 2.3 Random forests model

A frequently used machine learning model, random forests is a non-parametric, supervised ensemble machine learning technique that was first put forth by Breiman as an extension to address regression and classification issues (12, 13). Fisher's discriminant is employed as a linear classifier for every branch of the random forests, which is based on techniques that train a forests of binary decision trees. To separate the observations into two homologous groups, known as branches, the algorithm in an ensemble decision tree uses a binary arithmetic technique. This splitting procedure is repeated until the “tree” has fully grown (“node purity” is reached) (14).

Data analyses was done and the random forests model was created using the statistical program R version 4.4.4. Using the random Forest package in R software, random forests of the variables were created for prediction of the variable polyp; 500 trees size was specified to be used in order to produce reliable findings. The mean loss in accuracy and Gini index values were used to assess the significance of each individual variable. In comparison to variables with lower values, those with a greater mean decline in accuracy or Gini index value were deemed more important for the algorithm. The receiver operating characteristic (ROC) curve was drawn and the area under the curve (AUC) was calculated in order to determine the diagnostic power of the variables for the prediction of colonic polyps. 80% of patients were used for the creation of the model, while 20% were included in the model validation subgroup. External validation was performed on the specific dataset.

### 2.4 Method comparison

In order to evaluate if the Random Forests was a good choice to create a prediction model for colonic polyps, two other methods were also evaluated: a generalized linear model (GLM) and Support Vector Machine (SVM). Their performance was tested on the same datasets and compared to the initial algorithm. For SVM the e1071 and pROC packages in R were used, while for GLM stats and caret packages in R were used.

## 3 Results

### 3.1 Characteristics of patients' initial dataset

The initial data set included 164 patients, 89 with normal colonoscopies, 75 with colonic polyps. 46.34% male, with a mean aged of 62.54 years, 20% had diabetes and 33.53% were previously diagnosed with gastritis. The mean BMI was 29.55, the mean fasting glucose was 120.4 mg/dl, the mean hemoglobin value was 13.49 g/dl, with a mean platelet count of 254.3/L. Mean total cholesterol levels were 201.7 mg/dl, for LDL 119.4 mg/dl and HDL 53.17 mg/dl, triglycerides had a mean value of 149.66 mg/dl, while serum glutamic-oxaloacetic transaminase was 26.64UI/L Table 2.

**Table 2:** Characteristics of patients – initial dataset.

Variables	Patients *n* (total 164)	Min	1st Qu	Median	Mean	3rd Qu	Max
Sex							
Male	76						
Female	88						
Res							
Urban	102						
Rural	62						
Age		32	54	64	62.54	69	86
DZ							
Present	33						
Absent	131						
IMC		21	26	29	29.55	32	45
glic		62.2	98	106.5	120.4	123.5	350
Hb		4.8	12.5	13.6	13.49	14.9	18.2
plt		46.4	203.5	257	254.3	290	501
colest		84	177.5	200	201.7	231	322
LDL		45	96	120	119.4	140	232
HDL		21.9	42.73	51.75	53.17	60.7	120.15
Triglic		29.3	87.75	120.23	149.66	173.86	1,072.37
TGO		11	18	22	26.64	30	134.45
gastr							
Present	55						
Absent	109						
polip							
Present	75						
Absent	89						

For categorial data are *n* (number of patients). For numeric data: Min-minimum, 1st Qu-first quartile, Median, Mean, 3rd Qu-third quartile, Max-maximum.

When comparing data of the patients without polyps and those with polyps, only 2 variables showed a statistically significant difference (*p* < 0.05): sex and body mass index. More male patients were in the polyp group while the BMI in the group without polyps was lower than in the polyp group (median-27 vs. 31) Table 3.

**Table 3:** Characteristics of patients with and without polyps.

Variables	Total (*N*-164)	Group no polyps (*N*-89)	Group with polyps (*N*-75)	*p*-value
Sex				
Male-*n* (%)	76 (46.35)	32 (35.96)	44 (58.67)	0.0035
Female-*n* (%)	88 (53.65)	57 (64.04)	31 (41.33)	
Res				
Urban-*n* (%)	102 (62.19)	55 (61.80)	47 (62.67)	0.94
Rural-*n* (%)	62 (37.81)	34 (38.20)	28 (37.33)	
Age	64 (54,69)	64 (54,69)	63 (55.25, 71)	0.943
DZ-*n* (%)	33 (20.12)	20 (22.47)	13 (17.33)	0.413
IMC	29 (26, 32)	27 (26, 31)	31 (28, 34)	1.263 × 10^−7^
glic	106.5 (98, 123.5)	104.6 (97.5, 120)	108 (99.08, 125.2)	0.73
Hb	13.6 (12.5, 14.9)	13.4 (12.3, 14.5)	13.95 (12.8, 15)	0.068
plt	257 (203.5, 290)	264 (213, 305)	238 (198, 272.8)	0.136
colest	200 (177.5, 231)	208 (176, 238)	195.5 (178, 219.2)	0.18
LDL	120 (96, 140)	123 (97, 150)	118 (94.25, 135.75)	0.36
HDL	51.75 (42.73, 60.7)	53.47 (45.26, 61.21)	49.84 (41.25, 60)	0.09
Triglic	120.23 (87.75, 173.86)	125.98 (80.37, 188.4)	118.5 (90.14, 159)	0.38
TGO	26.64 (±15.71)	25.4 (±16.02)	28.12 (±15.31)	0.27
Gastr-*n* (%)	55 (33.54)	33 (37.08)	22 (29.33)	0.29

Data are expressed as *n* (%), mean ± standard deviation or median (Q1, Q3).

The dataset for external validation included 42 patients, 28 with normal colonoscopies, 14 with colonic polyps. 47.62% male, with a mean aged of 60.95 years, 7.14% had diabetes and 38.1% were previously diagnosed with gastritis. The mean BMI was 27.05, the mean fasting glucose was 104.6 mg/dl, the mean hemoglobin value was 13.54 g/dl, with a mean platelet count of 252.3/L. Mean total cholesterol levels were 195.1 mg/dl, for LDL 127.2 mg/dl and HDL 51.48 mg/dl, triglycerides had a mean value of 105.07 mg/dl, while serum glutamic-oxaloacetic transaminase was 23.64 UI/L Table 4.

**Table 4:** Characteristics of patients – external validation dataset.

Variables	Patients (total *N* = 42)	Min	1st Qu	Median	Mean	3rd Qu	Max
Sex							
Male	20						
Female	22						
Res							
Urban	25						
Rural	17						
Age		33	54.25	63.50	60.95	69	75
DZ							
Present	3						
Absent	39						
IMC		21	24	27	27.05	28	36
glic		75	85	96	104.6	109	370
Hb		10.8	12.62	13.55	13.54	14.7	16.1
plt		137	211.5	246	252.3	284.8	416
colest		111	171.2	190	195.1	215	287
LDL		59	103	123	127.2	147.5	206
HDL		27	41.75	50.5	51.48	60.25	98
triglic		17	64.25	102	105.07	147	203
TGO		11	17.25	20	23.64	25.75	76
gastr							
Present	16						
Absent	26						
polip							
Present	14						
Absent	28						

For categorial data are *n* (number of patients). For numeric data: Min-minimum, 1st Qu-first quartile, Median, Mean, 3rd Qu-third quartile, Max-maximum.

### 3.2 Random forests

The patients were randomly split 80–20 into a training set and a testing set. Using the training set a Random Forest model was created. The size was set at 500 trees and 3 variables were tried at each split.

While Random Forests don't require cross-validation to function (13), we used it to evaluate and tune the model. The “caret” package was used in R to specify 10-fold cross-validation. Different values for mtry (number of features considered at each split) and accuracy metric was used to evaluate the different mtry values. mtry of 2 or 3 proved to provide the highest accuracy, with minimal differences Table 5.

**Table 5:** Accuracy of mtry values.

Mtry	Accuracy
2	0.7199
3	0.7196
8	0.7140
14	0.7136

**Figure 2:**
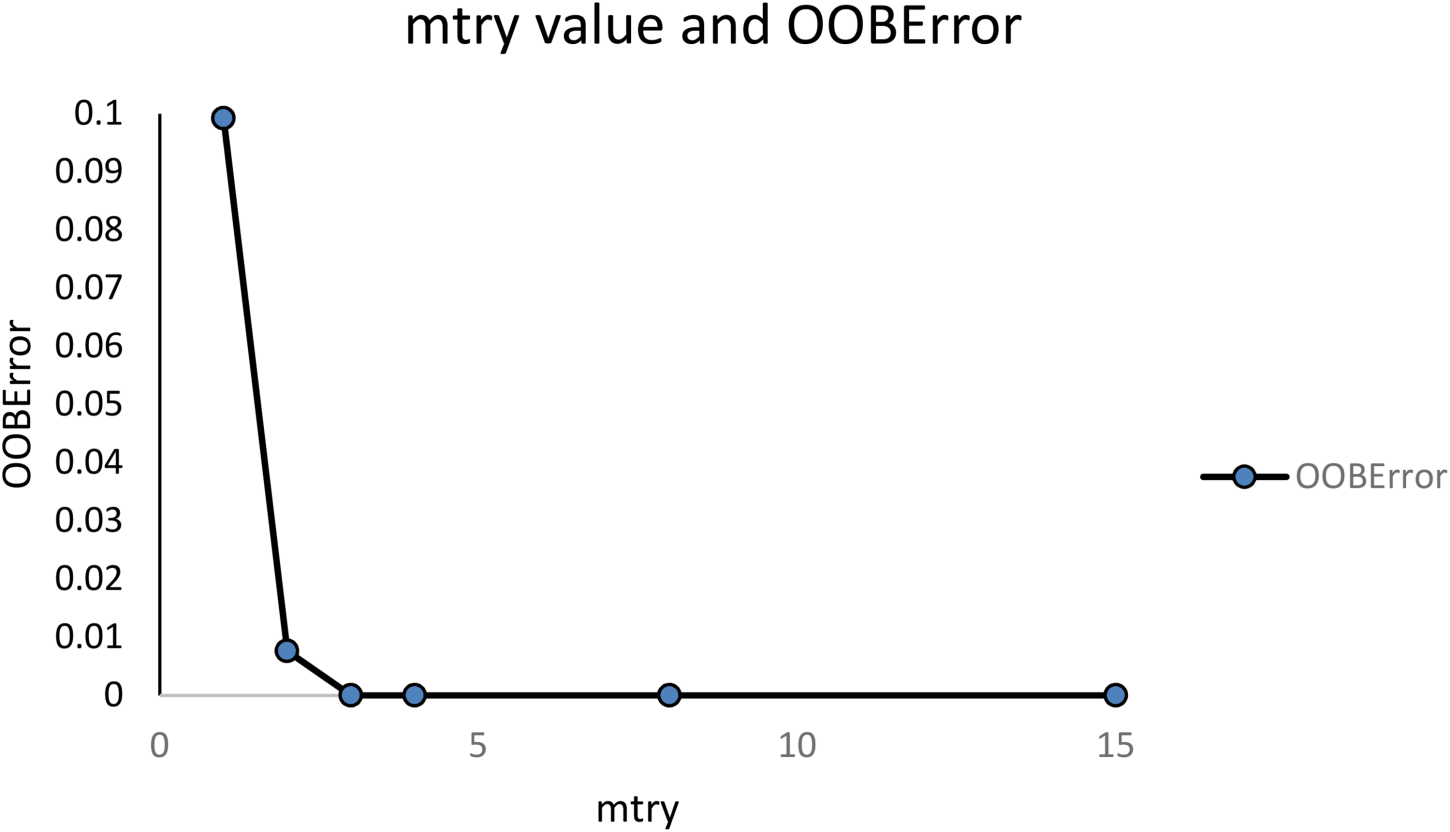
Out of bag error (OOBError) for different number of variables used at each split (mtry).

Using the “caret” package in R, fine tuning of mtry and number of trees (trees) was done to establish the best model and the OOBError (out of bag error) was used to select the best values. For an mtry = 1 the OOBError was 0.09923, for mtry = 2 the OOBError was 0.00763, while starting from mtry = 3 the error becomes 0. Figure 2 Mtry = 3 was selected for the algorithm.

For tunning the number of trees for the algorithm, different values were tried and 500 trees was associated with the lowest OOBError Table 6.

**Table 6:** Number of trees and out of bag error.

Trees	OOBError
100	25.95%
300	21.37%
**500**	**20.14%**
1,000	22.9%
5,000	22.14%

The number of trees generating the best OOBError is in bold.

### 3.3 Variable importance

**Figure 3:**
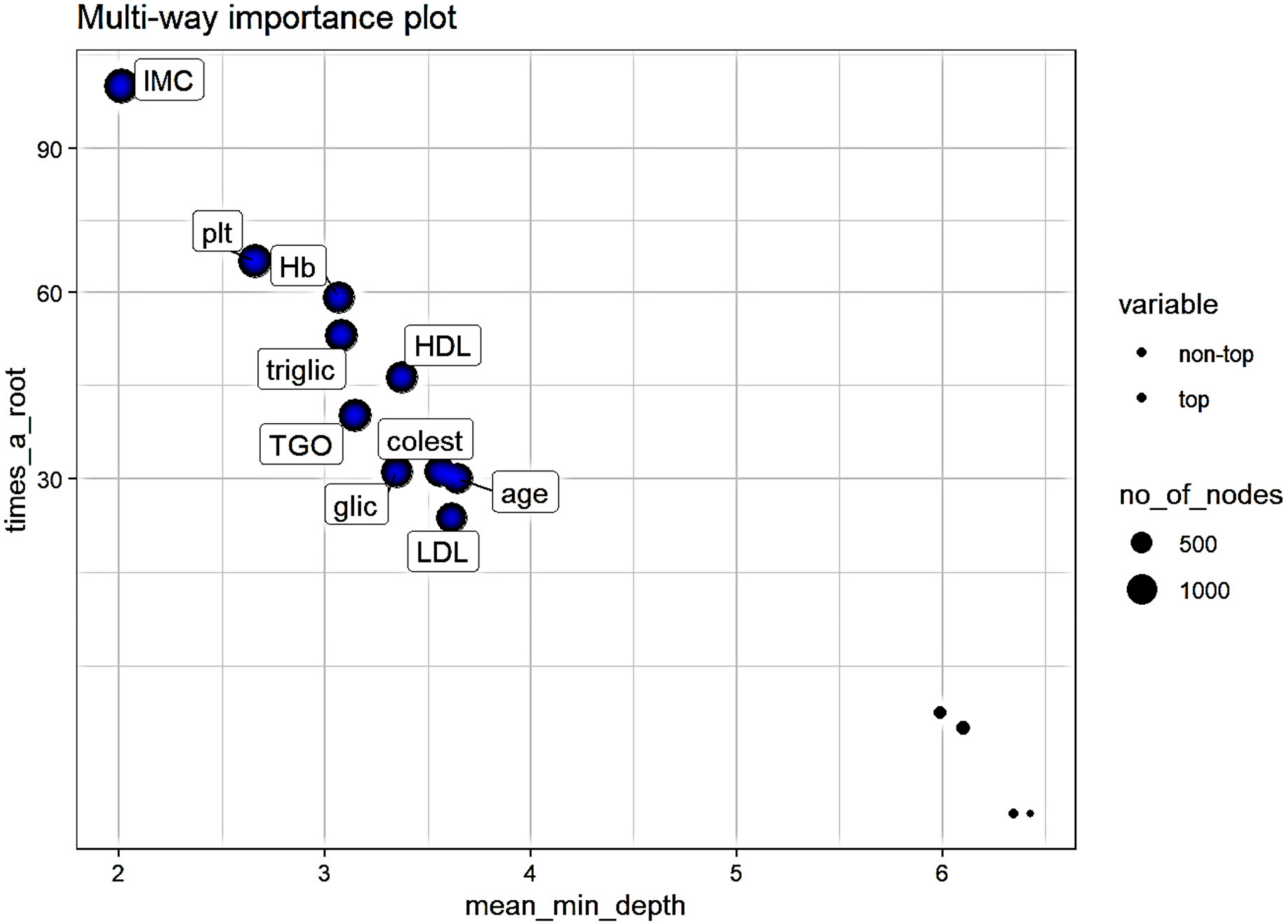
Multi-way importance plot of measures derived from the structures of the trees. Variables represented with bigger circles are used in more than 1000 nodes, top variables are represented with blue.

Analyzing the importance of the variables used while taking into consideration three measures derived from the structures of the trees (mean depth of first split of a variable, total number of nodes that split on that variable and the number of trees in which the variable splits the root) the variables with the most importance are (Figure 3):

- body mass index (IMC), mean minimum depth-2.01, number of nodes-1359, number of trees - 492
- platelets (plt), mean minimum depth-2.66, number of nodes-1266, number of trees - 470
- hemoglobin (Hb), mean minimum depth-3.06, number of nodes-1102, number of trees - 446
- triglicerides (triglic), mean minimum depth-3.08, number of nodes-1174, number of trees - 454
- glutamic-oxaloacetic transaminase (TGO), mean minimum depth-3.14, number of nodes-1172, number of trees – 455
- followed by: glycemia, HDL cholesterol, cholesterol, LDL cholesterol, and age.

**Figure 4:**
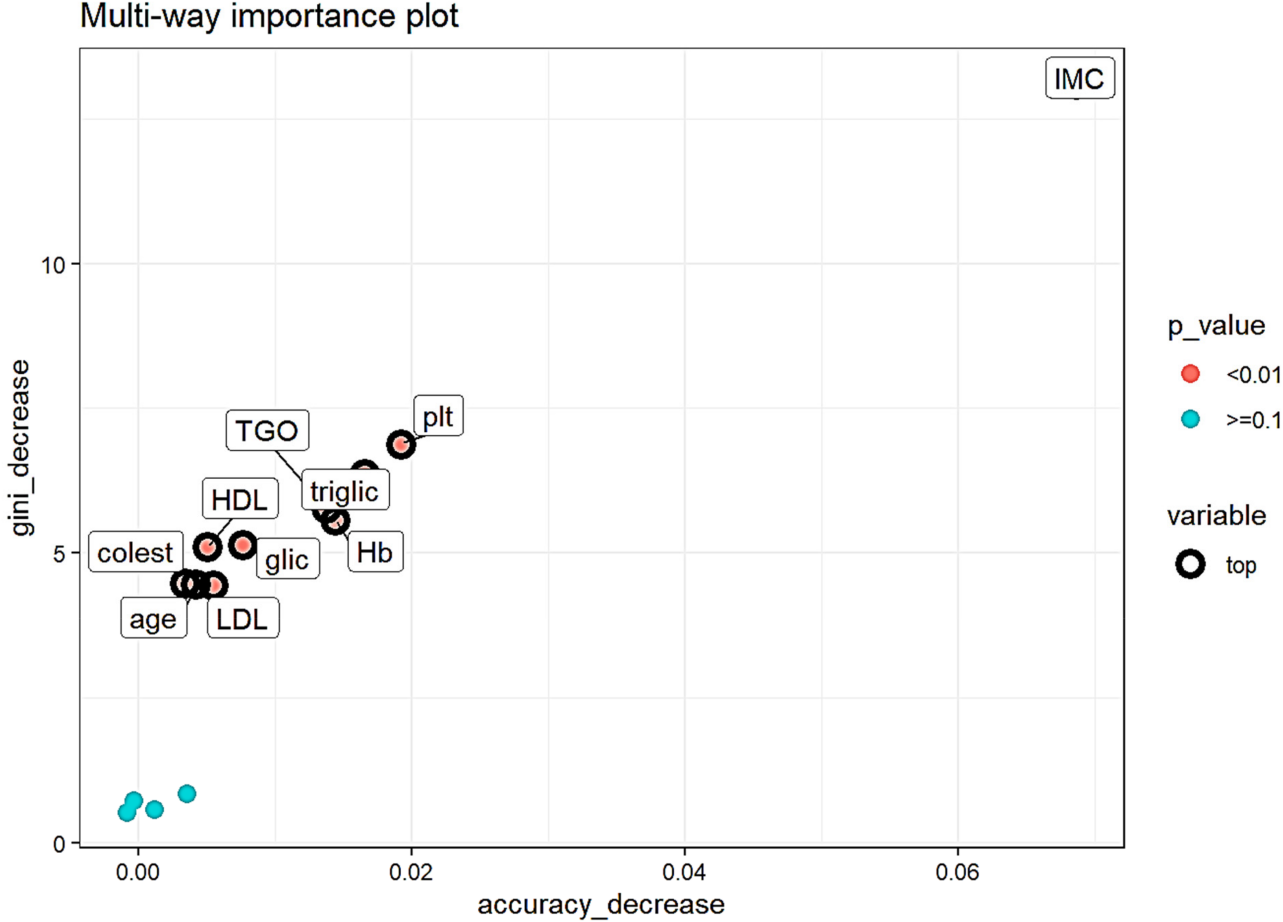
Multi-way importance plot of measures which take into consideration the predicting role of the variable. Top variables are represented with black circumference circles, variables with *p* < 0.01 are red, with *p* ≥ 0.1 are represented with light blue.

Analyzing the importance measures which take into consideration the role which the variable has in predicting (accuracy decrease, gini index decrease and *p*-value of a binomial distribution of the number of nodes which split on the variable assuming the variables are randomly used for splitting) (Figure 4) the top variables are (all with *p* < 0.01):

- body mass index (IMC), gini decrease- 13.09, accuracy decrease-0.07,
- platelets (plt), gini decrease-6.88, accuracy decrease-0.02,
- triglicerides (triglic), gini decrease- 6.36, accuracy decrease-0.02
- glutamic-oxaloacetic transaminase (TGO), gini decrease-5.77, accuracy decrease-0.01,
- hemoglobin (Hb), gini decrease-5.56, accuracy decrease- 0.01

**Figure 5:**
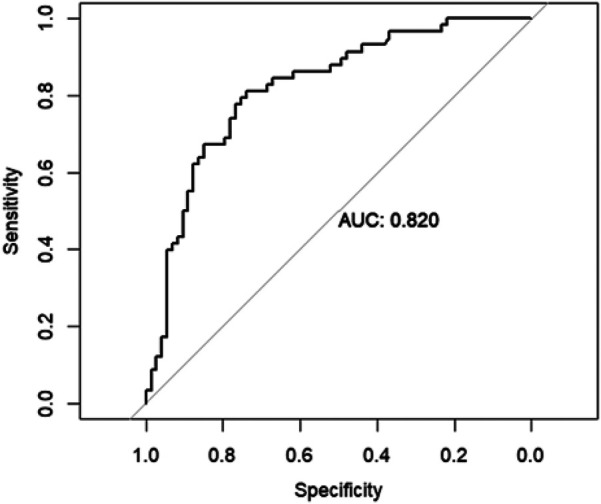
Area under the curve (AUC) of the receiver operating characteristics (ROC) of random forest model in the testing set.

To evaluate the performance of the random forest model, receiver operating characteristics (ROC) curve analysis, as it takes into consideration both sensitivity as well as specificity. AUC value was 0.820 (95% CI = 0.747–0.893), having a good discriminative power Figure 5.

**Figure 6:**
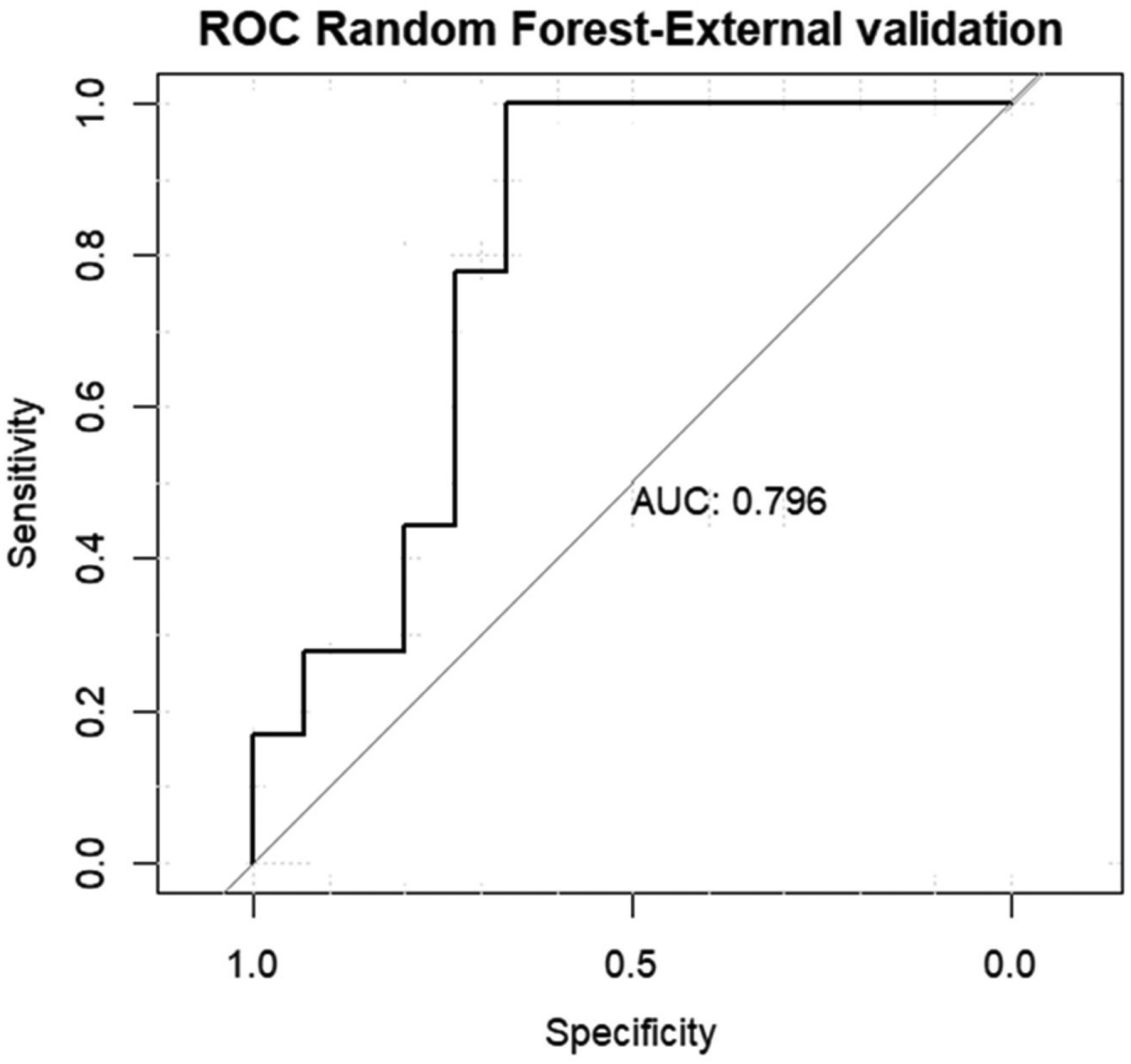
Area under the curve (AUC) of the receiver operating characteristics (ROC) of random forest model in the external validation data set.

For external validation AUC was 0.796 (95% CI = 0.718–0.851), The model's performance on the external validation dataset was slightly lower than on the internal validation dataset, which is expected. However, the drop in performance is minimal Figure 6.

### 3.4 Other methods

#### 3.4.1 Generalized linear model (GLM)

The initial GLM (binomial family) created included all the variables in order to predict the presence of polyps. In order to improve its performance manual down stepping based on *p*-values was done, the reduced model included only the following variables: DZ, IMC, plt, HDL and triglic. This model provided an AIC-190.76.

**Figure 7:**
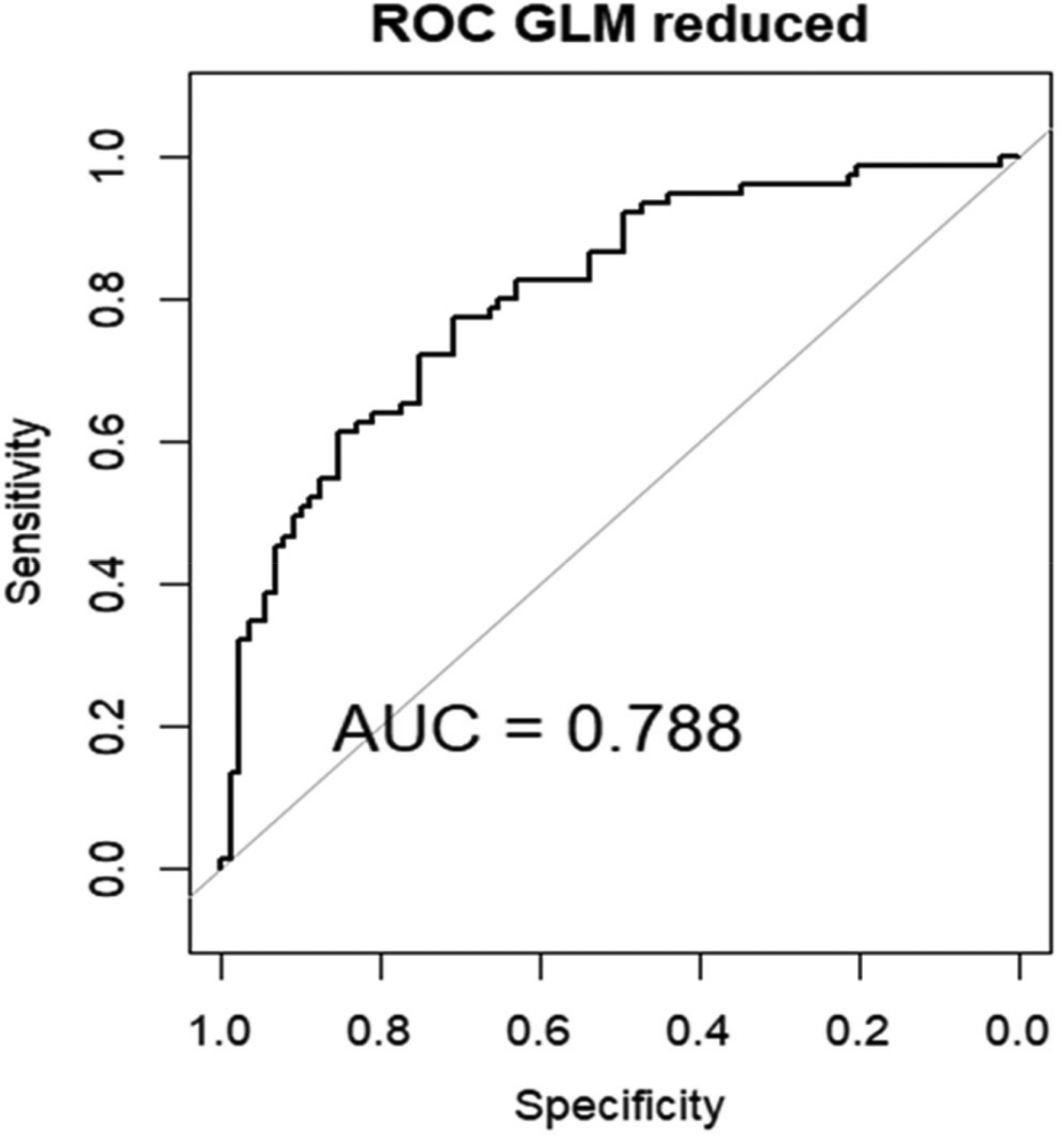
Area under the curve (AUC) of the receiver operating characteristics (ROC) of GLM in the internal validation data set.

The AUC of the ROC of this model, upon internal validation, was good: 0.788 Figure 7.

**Figure 8:**
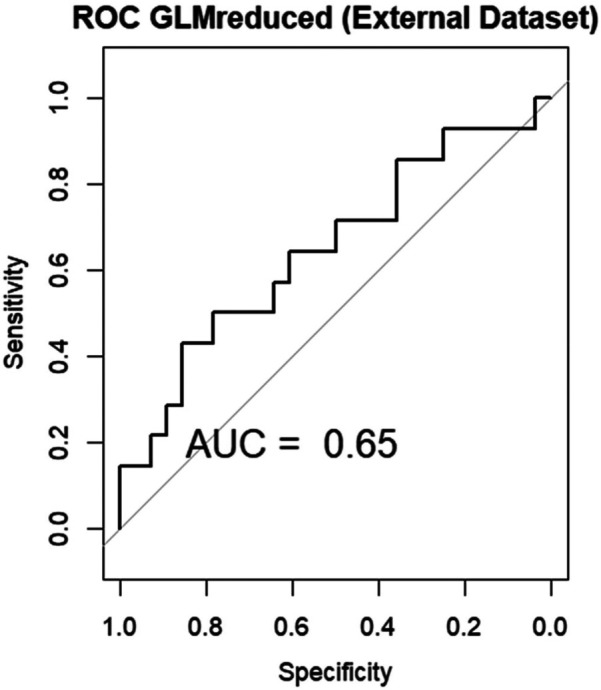
Area under the curve (AUC) of the receiver operating characteristics (ROC) of GLM in the external validation data set.

Analyzing the performance of the linear model on the external validation dataset, we observe an AUC of 0.65, showing a modest performance of the model on new data Figure 8.

#### 3.4.2 Support vector machine (SVM)

SVM are supervised learning models used for both classification and regression. In classification, SVM tries to find the hyperplane that divides best the data points of different classes in the feature space. The Radial Basis Function (RBF) kernel was used. Hyperparameter tuning was made using a grid search approach. The best combination found was cost = 10 and gamma = 1. Evaluation of the performance was made similar to the previous model, first on the internal validation dataset, then on the external validation dataset.

**Figure 9:**
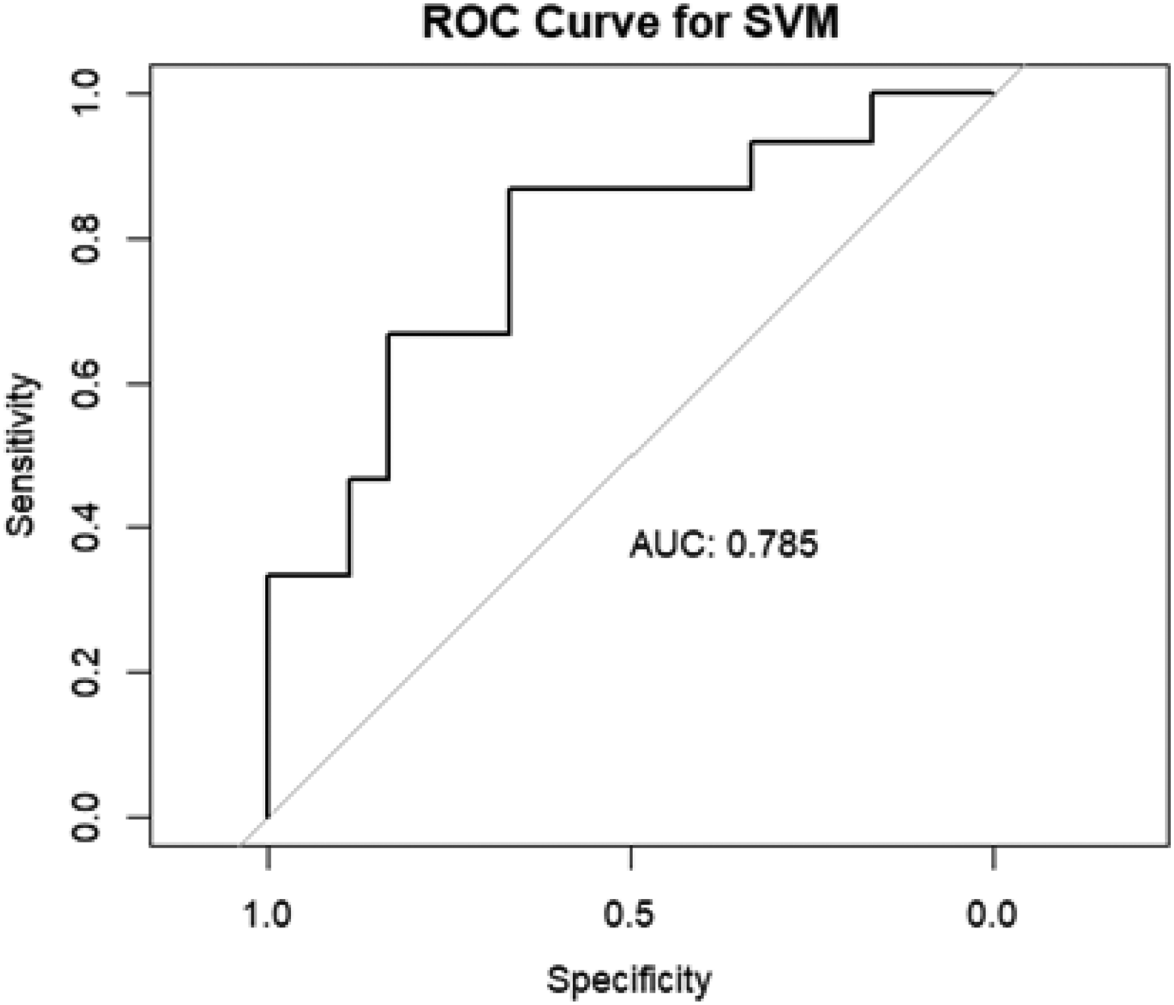
Area under the curve (AUC) of the receiver operating characteristics (ROC) of SVM in the internal validation data set.

The AUC of the ROC of this model, upon internal validation, was good: 0.785 Figure 9.

**Figure 10:**
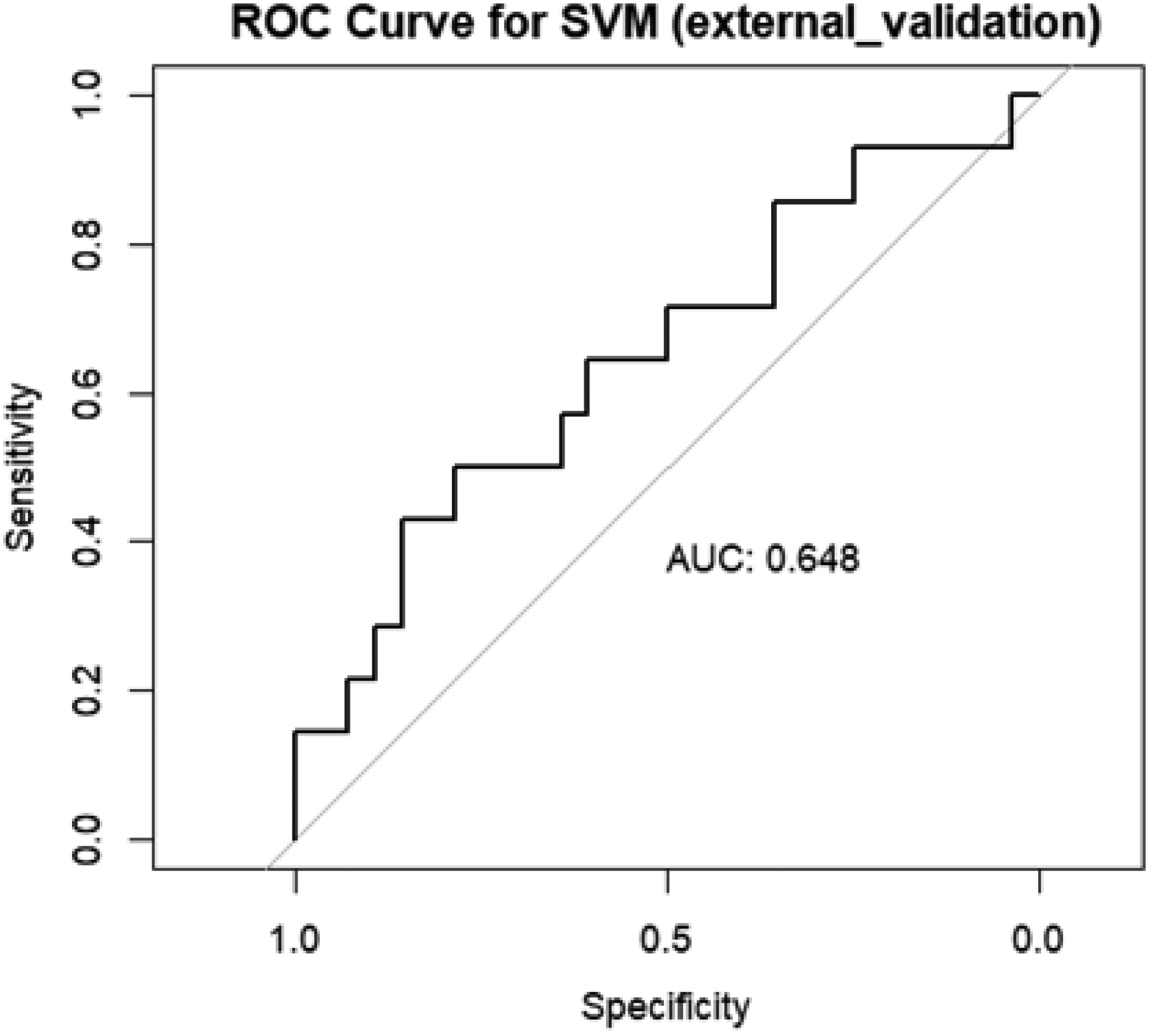
Area under the curve (AUC) of the receiver operating characteristics (ROC) of SVM in the external validation data set.

For external validation dataset, an AUC of 0.648 was obtained Figure 10.

## 4 Discussion

AI has a statistically significant positive influence on increasing the detection rate of colorectal polyps during colonoscopies (15). The application of AI algorithms is critical in reducing polyp miss rates in endoscopy. AI algorithms can analyze real-time images of the colon, highlighting alarming spots that humans may miss. This allows endoscopists to identify and remove polyps sooner, which is critical for preventing the development of colorectal cancer (16).

AI can analyse massive amounts of data from multiple sources and identify patterns in photos that indicate polyps (17). This allows AI to identify small changes in the mucosal surface that the human eye may overlook. Overall, these learning procedures have enhanced computer aided diagnostic systems (16, 18). AI algorithms rapidly scan the colon video footage and highlight suspicious areas that the endoscopist may have missed (19). This can reduce the polyp miss rate while increasing the detection rate, although it did increase the withdrawal time (20).

Computer aided diagnostic systems for colorectal polyps significantly increased adenoma detection rate or polyp detection rate with the use of different algorithms (21). The utility of AI in aiding the diagnosis of colorectal polyps during colonoscopy is questioned in certain studies (22–24). In a randomized controlled trial the computer aided diagnostic system showed a non-significant trend towards improving adenoma detection rate among patients undergoing screening or surveillance colonoscopy compared to high-definition white-light colonoscopy alone (25). The same lack of statistically significant increase in adenoma detection rate in real time endoscopies was found for using the GI Genius (Medtronic) module, an AI based computer aided diagnostic system (26). Also, no improvement in diagnosis was found when using AI in colonoscopies for patients with high risk of having colorectal lesions (27). When analyzing the clinical use of AI in colonoscopies, an improvement in lesion detection was seen for trainee endoscopists (28).

Li et al. developed a screening framework, Feature Interpretability Screening Framework, to identify patients at high risk for CRC. They used a combination of variables (sex, age, marital status), occult fecal test results, personal and family cancer history, gastrointestinal symptoms, obtained from a large patients' dataset (1,649,317) to train different artificial intelligence models in order to identify patients at high risk for CRC. The best performance was obtained by Naïve Bayes and SVM (highest sensitivity-0.779), Lasso had the highest specificity (0.868) and Logistic Regression -the highest AUC (0.859) (29). This study was done for CRC, on a large population database from a single medical center, we note that the Random Forests algorithm had an AUCs of 0.826, a value similar to our model, although a real comparison cannot be done between the studies, as they are trained for identifying patients with high risk of different lesions.

Zhang et al. constructed a ML extracellular vesicles based proteomics strategy model using a panel of 10 circulating protein markers which can predict well pre-malignant polyps and early stage CRC. The ML algorithms used, which provided excellent predictive power, were Naive Bayes, SVM, and Random Forest, having an AUC value that differentiate polyp from healthy, CRC from healthy, and CRC from polyp: 1, 0.97 and 0.94, respectively (30).

Random forests algorithms have been used to determine the relationship between gut microbiota and genetic factors in CRC. The model had good predicting potential of KRAS mutation status among CRC patients (AUC - 0.819), offering a potential new strategy for the precise treatment of CRC (31).

Artificial intelligence has also been used in differentiating adenomatous from non-adenomatous polyps on CT colonography. A Random Forest radiomics based model was developed and used for assisting radiologists in identifying polyp characteristics on CT colonography. The AI-assisted readings had higher accuracy, sensitivity, and specificity in selecting polyps eligible for polypectomy (32).

The development of clinical models of disease risk is the subject of several studies, and there are numerous relevant risk models available, such as those for colorectal cancer and coronary heart disease (33, 34). At the moment, colorectal cancer represents the basis for the majority of colorectal disease prediction models (35). Few colorectal polyp risk prediction models exist.

In this study we used a supervised learning model developed on easily obtainable and usually already available data for selecting patients with a high risk of being diagnosed with colonic polyps. Our algorithm had an AUC of 0.820. A study by Huang et al. developed a clinical predictive nomogram for the risk of a missed diagnosis of colorectal polyps in individuals based on multivariate analysis, the AUC being 0.747 (36). Their study was mainly focused on the necessity of performing a follow-up colonoscopy in certain patients at risk of having missed polyps during the initial procedure, as compared to our study which is focused on identifying which patients would benefit of a colonoscopy in order to identify and resect colorectal polyps. Ba et al. (37) developed a colorectal polyp prediction model using laboratory results, vital signs and demographic data from a big cohort of patients undergoing colonoscopies (5,426 patients). They included data similar to ours, but also more advanced lab tests (carcino embryonic antigen, hemoglobin A1c) which are not routinely done for the general population, making it harder to implement on a vast number of individuals. They tested 9 different ML methods and proved that, for their data, the adaptive boosting machine (AdaBoost) model had the best performance, providing an AUC = 0.675 on internal validation.

The incidence of colorectal polyps rises with age, according to numerous research. With every year of age gain, the risk of colorectal polyps increases by 1.03 times (38). The incidence rate of colorectal polyps rose with age and was higher in males than in females, according to the study of data of 327,785 colonoscopies performed in the US (39). In our study, the polyp group didn't have a statistically significant age difference, but it had more male participants than the no polyp group. Factors including bile acid synthesis, insulin-like growth factors, and estrogen receptor genes may be linked to females' decreased incidence of colorectal adenomatous polyps (40, 41). We noted the fact that the AI algorithm didn't include the sex variable in the top 10 most important variables, although it was statistically important, showing the completely different approach this algorithm has compared to more conventional statistical approaches regarding polyp prediction. Body mass index in the polyp group was higher than in the control group, which is consistent to other published studies (42).

Comparing the Random Forests algorithm with other two methods, generalized linear models and support vector machine, for our datasets, Random Forests provided better performance.

The model should be seen as a helpful tool for identifying unscreened individuals who are more likely to have precancerous lesions, rather than as a potential replacement for colonoscopies.

This research has a number of limitations. Data on eating habits, smoking, alcohol and drug use history, and family history, were not included, potentially excluding aspects associated with polyp formation. The medical records do not provide information on diet, while smoking and alcohol consumption information is not always realistically provided by patients. We also excluded patients with a family history of digestive tumors, as this is a separate risk factor, requiring attentive observation. This was a preliminary study, using a small number of patients. Only patients who had had a colonoscopy were included in the study population, which may not be representative of the general population. The study's retrospective design exposes it to selection bias, additionally the variables used to build the model were collected retrospectively, therefore it is uncertain how well the model performs in real time situations. The study is a single center study, the patients coming from a specific small region, which might have reduced the generalizability of our results. External validation was done on a small dataset, which contained data retrospectively obtained. Consequently, future research with bigger sample size would better evaluate our model (43, 44). Only the presence or absence of colonic polyps was assessed, without any other details. In the future, it would be useful to construct algorithms to also predict the presence of advanced adenomas or the size of the polyps as well as to create a calculator to determine the probability that asymptomatic people have colorectal polyps.

## 5 Conclusions

Colonic polyps have a risk of progressing into colonic cancer and their early diagnosis and removal might lead to a decrease in the incidence of colonic cancer. A random forest prediction model was developed using patient's demographic data, medical history and common blood tests results. This algorithm can foresee, with a high predictive power, the presence of colonic polyps.

## Data Availability

The data analyzed in this study is subject to the following licenses/restrictions: data will be made available on reasonable request from the authors. Requests to access these datasets should be directed to Mihaela-Flavia Avram - avram.mihaela@umft.ro.
